# C5aR1-positive adipocytes mediate non-shivering thermogenesis in neonatal mice

**DOI:** 10.1016/j.isci.2024.111261

**Published:** 2024-10-28

**Authors:** Huan-Yu Wang, Xue-Min Peng, Min Yang, Ying Weng, Xi Yang, Di Zhan, Qin Ning, Xiao-Ping Luo, Yong Chen

**Affiliations:** 1 Department of Pediatrics, Tongji Hospital, Tongji Medical College and State Key Laboratory for Diagnosis and Treatment of Severe Zoonotic Infectious Diseases, Huazhong University of Science and Technology, Wuhan 430030, China; 2 Hubei Key Laboratory of Pediatric Genetic Metabolic and Endocrine Rare Diseases, Wuhan 430030, China; 3 Research Group of Endocrinology & Metabolism, Key Laboratory of Vascular Aging, Ministry of Education, Tongji Hospital, Tongji Medical College, Huazhong University of Science and Technology, Wuhan 430030, China; 4 Division of Endocrinology, Department of Internal Medicine, Tongji Hospital, Tongji Medical College and State Key Laboratory for Diagnosis and Treatment of Severe Zoonotic Infectious Diseases, Huazhong University of Science and Technology, Wuhan 430030, China; 5 Department and Institute of Infectious Disease, Tongji Hospital, Tongji Medical College and State Key Laboratory for Diagnosis and Treatment of Severe Zoonotic Infectious Diseases, Huazhong University of Science and Technology, Wuhan 430030, China

**Keywords:** natural sciences, biological sciences, biochemistry, biochemical mechanism

## Abstract

Brown adipose tissue (BAT) plays an important role in maintaining body temperature in newborn mammals; however, its mechanisms remain poorly understood. Here, we report the identification of a special population of brown adipose tissue-derived stromal cells (ASCs) in neonatal mice that highly express CD45 and can be differentiated into adipocytes with lower thermogenic ability. These CD45^+^ adipocytes also characteristically contained complement C5a receptor 1(C5aR1) on the cell membrane. *C5ar1* deficiency in BAT resulted in an apparent immaturity of adipocytes and cold intolerance in neonatal mice. Mechanistically, loss of C5aR1 in these CD45^+^ brown adipocytes caused an increase in the secretion of plate factor four (PF4) from these cells, suppressing the maturity of neighboring brown adipocytes. Overall, our results indicated that the accumulation of C5aR1 positive brown adipocyte in neonatal BAT is essential for thermoregulation in newborn mice, which unveiled the regulatory mechanism of BAT-mediated thermogenesis in newborns.

## Highlights

• CD45-positive adipocytes contained C5aR1 on the cell membrane
• C5aR1-positive adipocytes emerged in brown adipose tissue in neonatal mice
• Loss of C5aR1 in adipocytes resulted in a reduction in BAT thermogenesis in neonatal mice
• PF4 secreted by C5aR1-positive adipocytes suppressed the maturity of brown adipocytes

## Graphical abstract

**Figure:**
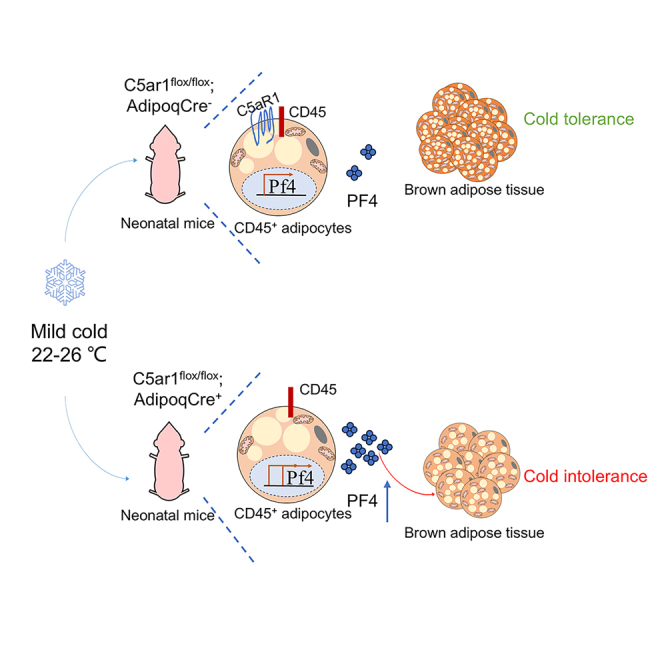

## Introduction

After birth, from intrauterine thermoneutrality to ambient temperature, rapid metabolic adaptations help protect the neonates against notable heat loss and hypothermia,^1^ which is critical for the survival of newborns. BAT-mediated non-shivering thermogenesis (NST) plays a major role in heat production in the neonates.^2^ The regulation of this process is largely and classically dependent on mitochondrial resided uncoupling protein 1 (UCP1) in brown adipocytes.^3^ It could uncouple the electron transfer chain from the oxidative phosphorylation process of ATP synthesis, allowing the production of heat as an end product instead of ATP by promoting proton leak.^4^ Newborns have a wealth of BAT in their bodies, which yet declines with age.^5^ Furthermore, an immaturity of BAT in newborns could also lead to a decrease in cold adaptation and therefore hypothermia.^1^ However, studies focusing on the regulatory mechanisms in the maturation of neonatal BAT are very limited.

In recent years, studies on the differences between adult and newborn BAT have reported that neonatal BAT morphologically contained smaller adipocytes with smaller but uniformly sized lipid droplets compared with adult BAT.^6^ However, newborns and juveniles displayed more BAT mass than adults, since BAT gradually vanished along with postnatal development and aging.^7^ Apart from these, the protein level of UCP1 and the mRNA expression levels of thermogenic genes, brown adipogenic genes, mitochondrial genes in neonatal BAT could be rapidly upregulated in response to cold in comparison with BAT of embryonic period (E19-20).^6^ All of these suggested that BAT activity was highest in the neonatal period among the entire life stage of mice, and further investigation is needed of dynamic processes of BAT from preadipocytes to maturity.

The heterogeneity of BAT has been demonstrated in the past few years that it contains visible subsets of brown adipocytes that own distinct functions and identity labels.^8^^,^^9^ Low and high thermogenic brown adipocyte subpopulations with low and high thermogenic activity, respectively, were identified in BAT of adult mice.^10^ Specifically, these low thermogenic brown adipocytes were distinct from the classical high thermogenic brown adipocytes, that they inhibit BAT-mediated thermogenesis by secreting ALDH1A1 to neighboring adipocytes on one hand and on the other hand promote thermogenesis by converting themselves to high thermogenic brown adipocytes.^8^ We doubt whether there are low thermogenic brown adipocytes in neonatal BAT as well and how they regulate BAT function, as it consists of a highly homogeneous population of brown adipocytes.

To investigate the unique recruitment of adipose tissue-derived stromal cells (ASCs) into BAT of neonatal mice, we performed single cell RNA sequencing (scRNA-seq) to explore total cells of stromal vascular fraction (SVF) from BAT of newborn mice. Interestingly, we found a subpopulation expressing both immune cell markers (*ptprc*, *Itgam*) and stem cell markers (*Itgb1*), which was rare in adult mice. These ASCs retained the expression of the pan-immune marker CD45, which was encoded by *ptprc*, so we termed them CD45^+^ ASCs. However, these ASCs exhibited a normal differentiation capacity into adipocytes, and mature CD45^+^ adipocytes expressed low thermogenic genes and high cytokine genes. Of note, knock down of *C5aR1*, a G-protein-coupled receptor (GPCR) that plays an important role in the development and functions of CD45^+^ adipocytes led to an inhibition of their thermogenic function and maturation. Specific depletion of *C5aR1* neonatal mice adipocytes caused an instant decrease in skin temperature during cold stimulation and an immaturity of brown adipocytes. Moreover, we showed that loss of C5aR1 signaling in these CD45^+^ brown adipocytes inhibited the thermogenic function of BAT by secreting plate factor four (Pf4) to suppress the maturity of neighbor brown adipocytes in neonatal mice. Together, we identified CD45^+^ brown adipocytes in the BAT of newborn mice, which were enriched with C5aR1, which plays an important role in the regulation of the thermogenic function of neonatal BAT.

## Results

### Identification and isolation of CD45^+^ ASCs in BAT of neonatal mice

**Figure 1:**
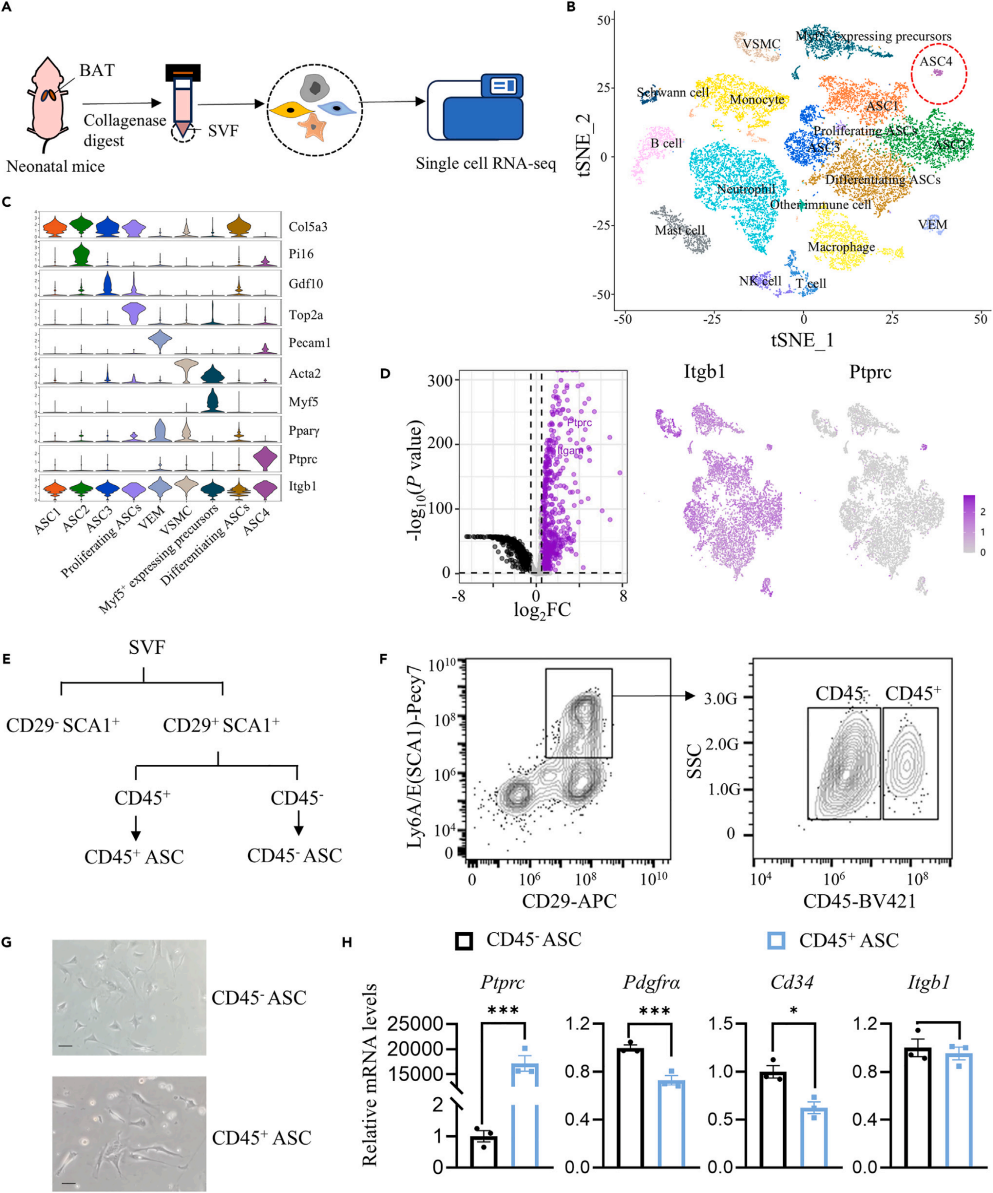
Identification of the CD45^+^ preadipocyte population in BAT of neonatal mice (A) Schematic diagram describing the experiment strategy to carry out single cell RNA seq of SVF from BAT from neonatal mice. (B) tSNE plot revealing SVF populations isolated from BAT of neonatal mice. (C) Normalized gene expression value as violin plots of selected cluster-specific genes from neonatal mice. (D) Volcano plot of the global gene expression profile of ASC1-3 and ASC4 (left) and tSNE plot showing colocalization of *Itgb1* and *Ptprc* in the ASCs and ASC4 clusters (right). (E) FACS gating strategy for the isolation of CD45^+^ ASCs by FACS. (F) CD45^+^ ASCs were isolated from CD29^+^ SCA1^+^ cells with CD45^+^. (G) CD45^−^ and CD45^+^ ASCs had adherent growth capacity, morphology of fibroblast-like cells. Scale bars, 10μm. (H) RT-qPCR of CD45^−^ and CD45^+^ ASCs sorted by FACS. CD45^+^ ASCs markers, *Ptprc*, adipose progenitor markers: *Pdgfrα*, *Cd34*, and *Itgb1* (*n* = 3). Statistical significance was assessed by two-tailed Student’s t test. Data are represented as mean ± SEM. ∗ ≤0.05, ∗∗ ≤0.01, ∗∗∗ ≤0.005.

To characterize the heterogeneity of BAT-derived stromal cells and immune cells from neonatal mice at single cell resolution, we performed single cell RNA-sequencing (scRNA-seq) analysis on total SVF cells from BAT from newborn mice (Figure 1A). The BAT pads of 6–7 neonatal mice were subjected to a scRNA-seq, which was repeated three times in total. At least 19 cell groups were identified with unsupervised clustering of gene expression profiles based on a t-distributed stochastic neighbor embedding (t-SNE) map (Figures 1B and S1A). We detected eight populations of immune cells, including monocytes, macrophages, neutrophils, B cells, T cells, natural killer cells (NK), mast cells, dendritic cells (Figure S1B). Apart from immune clusters, three clusters of classical ASC1-3 were identified with expression of adipocyte progenitor cell markers *Pdgfrα*, *Cd34*, and lymphocyte antigen 6 complex locus A (*Ly6a*) but without expression of adipogenic marker *Fabp4*^11^ (Figure S1C). Proliferating ASCs expressed high levels of proliferative markers, such as *Top2a* and *Birc5*,^12^ while differentiating ASCs expressed high levels of adipogenic markers, such as *Pparγ* and *Fabp4*^13^ (Figure S1C). Furthermore, My5^+^ precursors were detected by scRNA-seq, which may be attributed to an immaturity of neonatal BAT.^14^ The most prominent feature was the identification of an additional cluster in neonatal BAT SVF, which was called ASC4 (Figure 1B and S1A). This cluster of cells highly expressed both *Itgb1* (*CD29*), a thermogenic marker,^15^ and *Ptprc (CD45)*, an immune cell marker, but did not express *CD34*, which is an adipocyte progenitor cell marker (Figures 1C and S1C). A recent study uncovered that human CD34^−^ adipocyte progenitors (CD34^−^, CD29^+^) could differentiate into thermogenic adipocytes with an enrichment of *Ucp1*.^16^ Compared to ASC1-3, these ASC4 cells showed higher mRNA expression levels of immune markers including *F4/80* (*Adgre1*), *Lgals3*, and *CD11b* (*Itgam*), and higher expression levels of cytokines, such as *Ccl3*, *Ccl4*, *Ccl6*, and *Ccl9* (Figure S1D). To isolate ASC1-3 and ASC4 from SVF, we found two highly enriched cell membrane markers *Ptprc* and *Itgb1* (Figure 1D).

Consequently, we distinguished ASC1-3 and ASC4 derived from SVFs by using their markers including CD29, Sca1, and CD45 and named ASC1-3 as CD45^−^ ASCs, ASC4 as CD45^+^ ASCs. The ASCs were first isolated from neonatal BAT SVF using CD29 and Sca1. Next, the CD45^−^ and CD45^+^ ASCs were separated using CD45 (Figures 1E and 1F). The CD45^+^ ASCs were approximately 18.2% of the ASCs and the CD45^−^ ASCs were approximately 4.5 times more than the CD45^+^ population (Figure S1E). After cell culture for 48 h, we found that both CD45^−^ and CD45^+^ ASCs showed morphological characteristics as stem cells but not as immune cells (Figure 1G). After another 5 days of culture, both CD45^−^ and CD45^+^ ASCs proliferated and reached a confluency of 100% (Figure S1F). Furthermore, we examined the difference in certain gene expression between these two cell populations by RT-qPCR. Undoubtedly, a higher expression level of *Ptprc* was confirmed in CD45^+^ ASCs than CD45^−^ ASCs (Figure 1H). Moreover, the adipocyte progenitor markers *CD34* and *Pdgfrα* were also expressed in CD45^+^ ASCs, which were lower than in CD45^−^ ASCs. However, the expression of the thermogenic marker *Itgb1* was identical between CD45^−^ and CD45^+^ ASCs (Figure 1H). These results illustrated that CD45^+^ ASCs existed in the BAT of newborn mice and could proliferate *in vitro*.

### The *in vitro* differentiated CD45^+^ adipocytes were distinct from classical brown adipocytes

**Figure 2:**
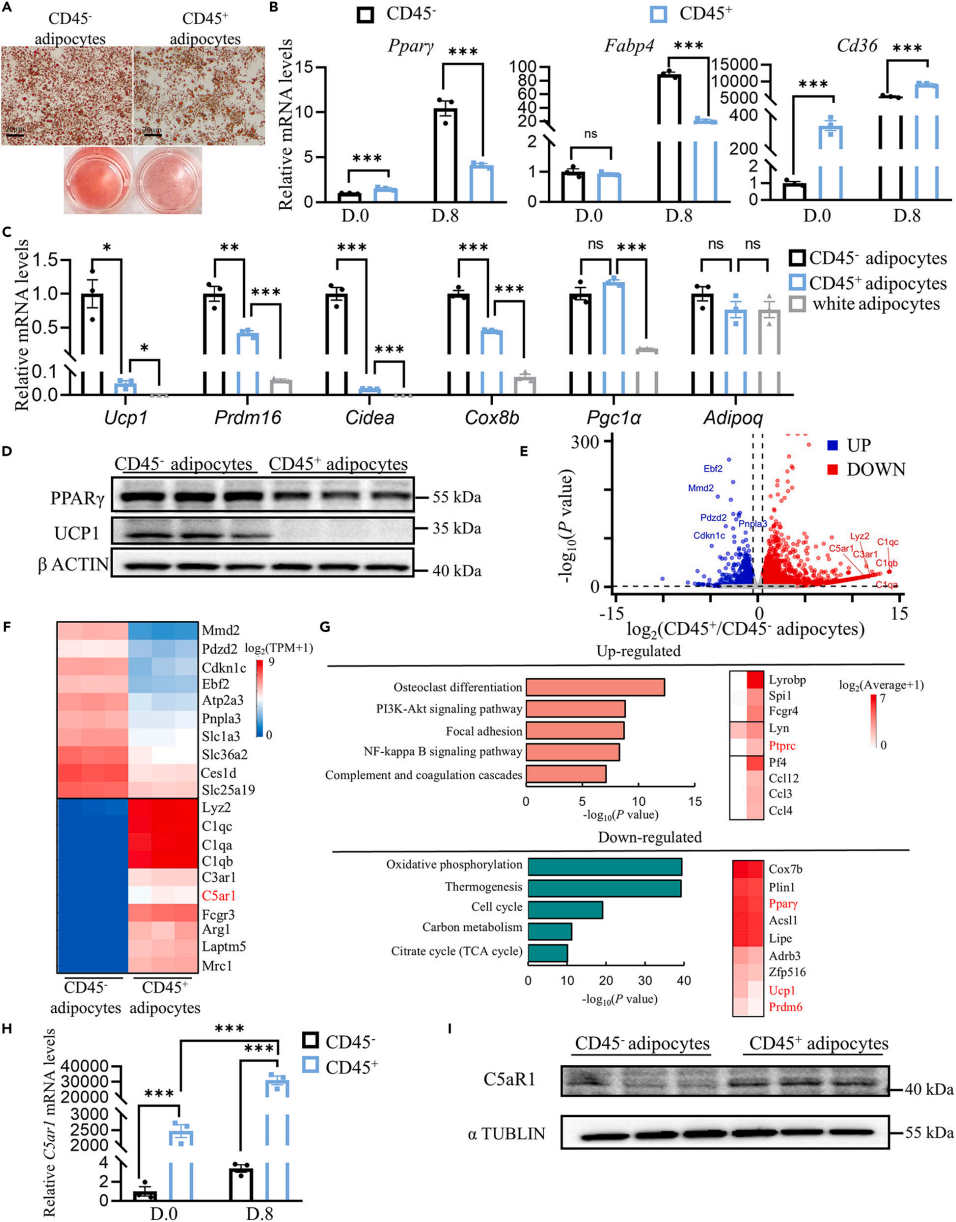
*In vitro* differentiated CD45^+^ adipocytes were distinct from classical brown adipocytes (A) Oil red O staining of adipogenic differentiation of isolated CD45^−^ and CD45^+^ ASCs *in vitro*. Scale bars, 20μm. (B) mRNA expression of the adipogenic differentiation of isolated CD45^−^ and CD45^+^ ASCs *in vitro*. (*n* = 3). (C) mRNA expression of the indicated genes in differentiated adipogenic cells in (A) and white adipocytes (*n* = 3). (D) Immunoblotting for UCP1 and PPARγ in differentiated cells in (A). (E) RNA-seq of CD45^−^ and CD45^+^ adipocytes. Volcano plot of the global gene expression profile (*n* = 3). (F) Top 10 genes upregulated and downregulated. (G) (Top) upregulated KEGG pathways and typical genes. (Bottom) downregulated KEGG pathways and typical genes. (H) Expression of *C5ar1* mRNA in CD45^−^ and CD45^+^ ASC and adipogenic differentiated CD45^−^ and CD45^+^ adipocytes. (I) Immunoblotting for C5aR1 in adipogenic differentiated CD45^−^ and CD45^+^ adipocytes. Statistical significance was assessed by two-tailed Student’s t test. Data are represented as mean ± SEM. ∗ ≤0.05, ∗∗ ≤0.01, ∗∗∗ ≤0.005.

Next, CD45^+^ and CD45^−^ ASCs were induced and differentiated into adipocytes after reaching confluency (Figure S2A). Both CD45^+^ and CD45^−^ ASCs showed a morphological change with an accumulation of lipid drops during differentiation (Figure S2B). We also found co-expression of CD45 and CD29 in both CD45^+^ ASC and CD45^+^ adipocytes (Figure S2C). A tiny decrease in lipid accumulation was observed in CD45^+^ adipocytes compared to that in CD45^−^ adipocytes (Figures 2A and S2D). Furthermore, we examined changes in adipogenic gene expression in CD45^+^ and CD45^−^ adipocytes. We found that CD45^+^ ASCs showed higher expression levels of *Pparγ* and *CD36* than CD45^−^ ASCs, while *Fabp4* expression was similar between CD45^+^ and CD45^−^ ASCs at day 0, suggesting that CD45^+^ ASCs may have similar differentiation ability as CD45^−^ ASCs. As expected, the expression of *Pparγ* and *Fabp4* increased in CD45^−^ cells after differentiation. But to our surprise, CD45^+^ adipocytes had lower expression level of *Pparγ* and *Fabp4* but a higher expression level of *CD36* than CD45^−^ adipocytes (at day 8) (Figure 2B). Moreover, CD45^+^ adipocytes showed significantly lower mRNA levels of thermogenic genes (*Ucp1*, *Prdm16)*, brown fat selected genes (*Cidea*, *Cox8b*) and all genes mentioned previously have higher expression levels in CD45^+^ adipocytes than white adipocytes (Figure 2C). In addition, CD45^+^ adipocytes also showed lower expression of PPARγ and UCP1 on protein level compared to CD45^−^ adipocytes (Figure 2D).

We wondered about the differences in the gene profiles between CD45^+^ and CD45^−^ adipocytes, so an RNA-seq was performed. Analysis in the volcano plot (Figure 2E) and the heatmap (Figure S2E) revealed that there were 4024 genes with different expression levels in CD45^+^ and CD45^−^ adipocytes, among which 2401 genes were upregulated and 1623 genes were downregulated in CD45^+^ adipocytes compared to that in CD45^−^ adipocytes. We selected the top 10 genes, which were up or downregulated, respectively (Figure 2F). The genes most upregulated in CD45^+^ adipocytes were immune cell markers (*Lyz2*, *C1qc*, *C1qa*, *C1qb*, *Fcgr3*, *Mrc1*), complement receptors (*C5aR1*, *C3aR1*)^17^ and regulation factors (*Arg1*, *Laptm5*). Kyoto Encyclopedia of Genes and Genomes (KEGG) pathway analysis showed that osteoclast differentiation, the PI3K-Akt signaling pathway, focal adhesion, the NF-κB signaling pathway, complement and coagulation cascades were significantly regulated up, while metabolic related pathways including oxidative phosphorylation, thermogenesis, cell cycle, carbon metabolism, citrate cycle (TCA cycle) were significantly negatively regulated in CD45^+^ adipocytes compared to CD45^−^ adipocytes. Besides, CD45^+^ adipocytes also showed as higher expression level of chemokines, such as *Pf4*, *Ccl3*, *Ccl4*, *Ccl12*, than CD45^−^ adipocytes (Figure 2G). Overall, it was demonstrated that the new identified CD45^+^ ASCs population could differentiate into unique mature CD45^+^ adipocytes, which may play a role in the regulation of BAT function in neonatal mice.

### Loss of *C5ar1* in adipocytes attenuated thermogenesis in newborn mice

Among the top upregulated genes in CD45^+^ adipocytes (Figure 2F), *C5ar1* and *C3aR1* were the only two GPCR, which play an important role in metabolic regulation and cytokine release. Subsequent analysis revealed alterations in the expression levels of *C5ar1* and C3aR1 pre- and post-differentiation of CD45^+^ adipocytes. It was observed that the expression of *C5ar1* was significantly elevated in CD45^+^ ASCs compared to CD45^−^ ASCs, with a 2500-fold increase, and showed a further 10-fold rise by day 8 following CD45^+^ adipocyte differentiation. Similarly, C3aR1 expression was found to be 200-fold higher in CD45^+^ ASCs compared to CD45^−^ ASCs, and exhibited a 2-fold increase by day 8 post CD45^+^ adipocyte differentiation (Figures 2H and S2F). Spectacularly, it was revealed that almost all CD45^+^ ASCs expressed the C5aR1 protein on the cell membrane by fluorescence-activated cell sorting (FACS) analysis (Figures S3A and S3B), which was consistent with the results of western blot (Figure 2I). Next, we conducted a study in which we examined and contrasted the proportion of CD45^+^CD88^+^ ASC within Sca1^+^ cells obtained from the SVF of BAT in neonatal mice, as well as BAT, inguinal (ING), and epididymal (EPI) adipose tissues in both young and aged mice. Utilizing FACS analysis, we observed a notably high presence of CD45^+^CD88^+^ ASC specifically in BAT. Furthermore, our findings indicated a decrease in the number of CD45^+^ ASC with aging, with the quantity of CD45^+^ ASC in BAT showing an age-related pattern (Figures S3C–S3E). Therefore, we hypothesized that C5aR1 might participate in the development and maturation of CD45^+^ adipocytes.

**Figure 3:**
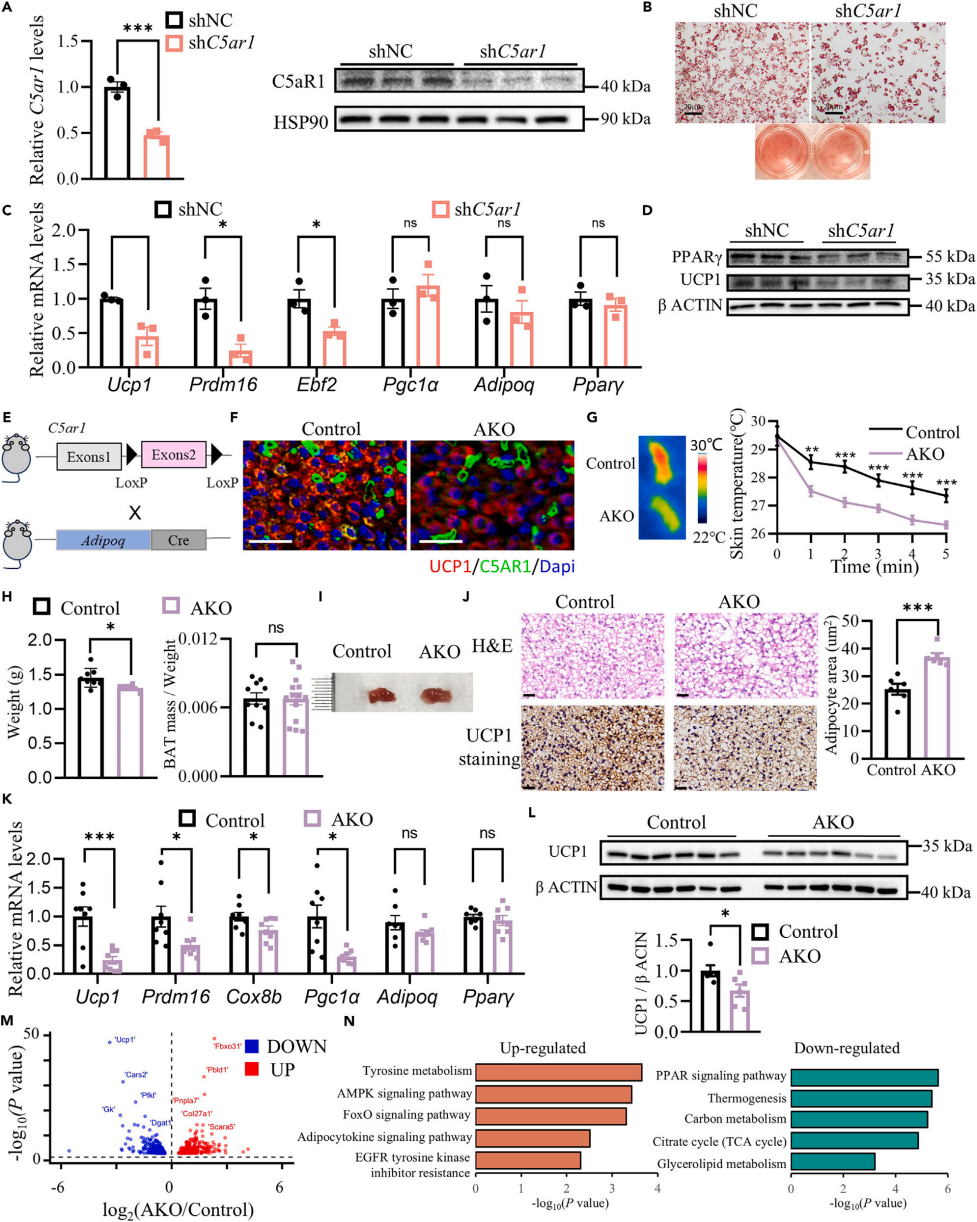
The loss of the *C5ar1* gene in adipocytes during the perinatal period decreased the thermogenesis ability of newborn mice (A) Administration of shRNA-*C5ar1* in CD45^+^ adipocytes significantly suppressed *C5ar1* expression compared to the control group. (Left) RT-qPCR for *C5ar1* in shNC and sh*C5ar1* CD45^+^ adipocytes. (Right) Immunoblotting for C5aR1 in shNC and sh*C5ar1* CD45^+^ adipocytes (*n* = 3). (B) Oil red O staining of shNC and sh*C5ar1* CD45^+^ adipocytes. Scale bars, 20μm. (C) The mRNA expression of the indicated genes of *C5ar1* knockdown differentiated CD45^+^ adipocytes (*n* = 3). (D) Immunoblotting for UCP1 and PPARγ of *C5ar1* knockdown differentiated CD45^+^ adipocytes (*n* = 3). (E) *C5ar1* AKO mice (*C5ar1*^flox/flox^; *Adipoq*Cre^+^) were generated by breeding *C5ar1*^flox/flox^ mice with *Adiponectin*-Cre mice. (F) Immunofluorescence staining of UCP1 and C5aR1 in the BAT of the control and *C5ar1* AKO neonatal mice. Scale bars, 50μm. (G) (left)Infrared thermo-imaging 5 min after the beginning of cold challenge. (Right) back skin temperature during cold challenge (22° C) (control, *n* = 8 AKO, *n* = 7). (H) Body weight and BAT/body weight ratio in control and *C5ar1* AKO neonatal mice. (I) Gross appearance of the interscapular BAT of the control and *C5ar1*AKO neonatal mice. Scale bars, 1mm. (J) H&E (Top) and UCP1 immunohistochemical staining (bottom). Scale bars, 20μm. BAT adipocyte sizes of control and *C5ar1* AKO neonatal mice (right). (K) Relative mRNA expression of indicated genes of BAT from control and *C5ar1* AKO neonatal mice (*n* = 8). (L) Immunoblotting for UCP1 of BAT from control and *C5ar1* AKO neonatal mice (*n* = 6). (M) RNA-seq of BAT from control and *C5ar1* AKO neonatal mice. Volcano plot of the global gene expression profile (*n* = 3). (N) Upregulated KEGG pathways and downregulated KEGG pathways. Statistical significance was assessed by two-tailed Student’s t test. Data are represented as mean ± SEM ∗ ≤0.05, ∗∗ ≤0.01, ∗∗∗ ≤0.005.

To investigate the role of C5aR1 in CD45^+^ adipocytes, the knockdown of *C5ar1* in CD45^+^ ASCs was performed using shRNA. A decrease in C5aR1 expression was verified in these cells both the mRNA and the protein level (Figure 3A). Oil red O staining revealed a decrease in lipid accumulation with *C5ar1* deficiency in CD45^+^ adipocytes (Figures 3B and S3F). Along with *C5ar1* deficiency, thermogenesis-related genes such as *Ucp1*, *Prdm16*, *Ebf2* were significantly downregulated in these cells. Moreover, genes related to adipogenic differentiation, such as *Adipoq* and *Pparγ* did not show any change in their expression after *C5ar1* deficiency (Figure 3C). In addition, the elimination of *C5ar1* led to a decrease in protein levels of PPARγ and UCP1 in these cells, which play a key role in thermogenesis (Figure 3D). These results indicated that C5aR1 was a critical molecule in the regulation of maturation and thermogenic function of CD45^+^ adipocytes.

To investigate the role of C5aR1 in CD45^+^ adipocyte-mediated thermogenesis in neonatal mice, we generated adipocyte-specific *C5ar1* KO mice (*C5ar1* AKO) by crossing *adiponectin*-Cre mice with *C5ar1*-Flox mice (Figure 3E), and mice on postnatal day 1 were used for further studies. The deletion of *C5ar1* in BAT was validated through RT-qPCR analysis (Figure S3g), protein level assessment via western blot (Figure S3H), and immunofluorescence colocalization analysis, which revealed that nearly all Ucp1^+^ adipocytes exhibited a reduction in C5aR1 expression (Figures 3F and S3I). To further examine the thermogenic function of BAT of *C5ar1* AKO mice, we exposed these mice from 30° C to room temperature (22° C–26° C) for 5 min. Compared to control mice, *C5ar1* AKO neonatal mice showed a faster decrease in body temperature (Figure 3G). The *C5ar1* AKO neonatal mice showed significantly decreased body weight (BW) but no change in the BAT mass/weight ratio compared to the control neonatal mice (Figure 3H). The BAT of the *C5ar1* AKO neonatal mice appeared lighter brown color compared to the control mice that presented a typical brown color (Figure 3I). An abundant accumulation of lipid droplets in the BAT of *C5ar1* AKO neonatal mice by hematoxylin and eosin (H&E) staining (Figure 3J). In general, deletion of *C5ar1* in adipocytes of neonatal mice could lead to weight loss and lower BAT thermogenesis.

Next, we detected the expression of thermogenic genes at both the mRNA and the protein level in BAT of *C5ar1* AKO neonatal mice. The mRNA expression of thermogenic genes, such as *Ucp1*, *Prdm6*, *Cox8b*, *Pgc1α*, and batokines, such as *Pltp*, decreased significantly, while no change in adipogenic genes mRNA expression, such as *Adipoq* and *Pparγ*, were detected in BAT of *C5ar1* AKO neonatal mice compared to control mice (Figures 3K and S3J). The protein level of UCP1 was significantly decreased in these AKO mice verified by western blotting (Figure 3L) and immunohistochemical staining (Figure 3J). We performed RNA-seq of BAT from control and *C5ar1* AKO neonatal mice, and detected 277 genes upregulated and 289 genes downregulated in the BAT of *C5ar1* AKO neonatal mice compared to control mice (Figures 3M and S3K). KEGG pathway analysis of transcriptome data revealed that the PPAR signaling pathway, thermogenesis, carbon metabolism, citrate cycle (TCA cycle) and glycerolipid metabolism were significantly negatively regulated, while tyrosine metabolism, the AMPK signaling pathway and the FoxO signaling pathway were significantly positively regulated in *C5ar1* AKO mice compared to control mice. Downregulation of PPAR signaling suggested an immaturity of BAT in *C5ar1* AKO neonatal mice that could lead to a decrease in metabolic level and thermogenic ability (Figure 3N). We examined the *C5ar1*, thermogenesis gene expression, and UCP1 protein levels in the inguinal white adipose tissue of male *C5ar1* AKO mice at 8 weeks of age. Our results indicated that there were no significant differences in the levels of *C5ar1* and thermogenesis mRNA in the inguinal white adipose tissue between the control group and the *C5ar1* AKO mice. (Figure S3I). UCP1 protein levels of inguinal white adipose tissue were not changed in *C5ar1* AKO mice either (Figure S3M). Overall, these data suggested that the adipocytes’ C5aR1 is necessary for the maturation of BAT and its mediated thermogenesis in neonatal mice.

### *C5ar1* knockdown in CD45^+^ brown adipocytes promoted the release of PF4 to inhibit the maturation of brown adipocytes

**Figure 4:**
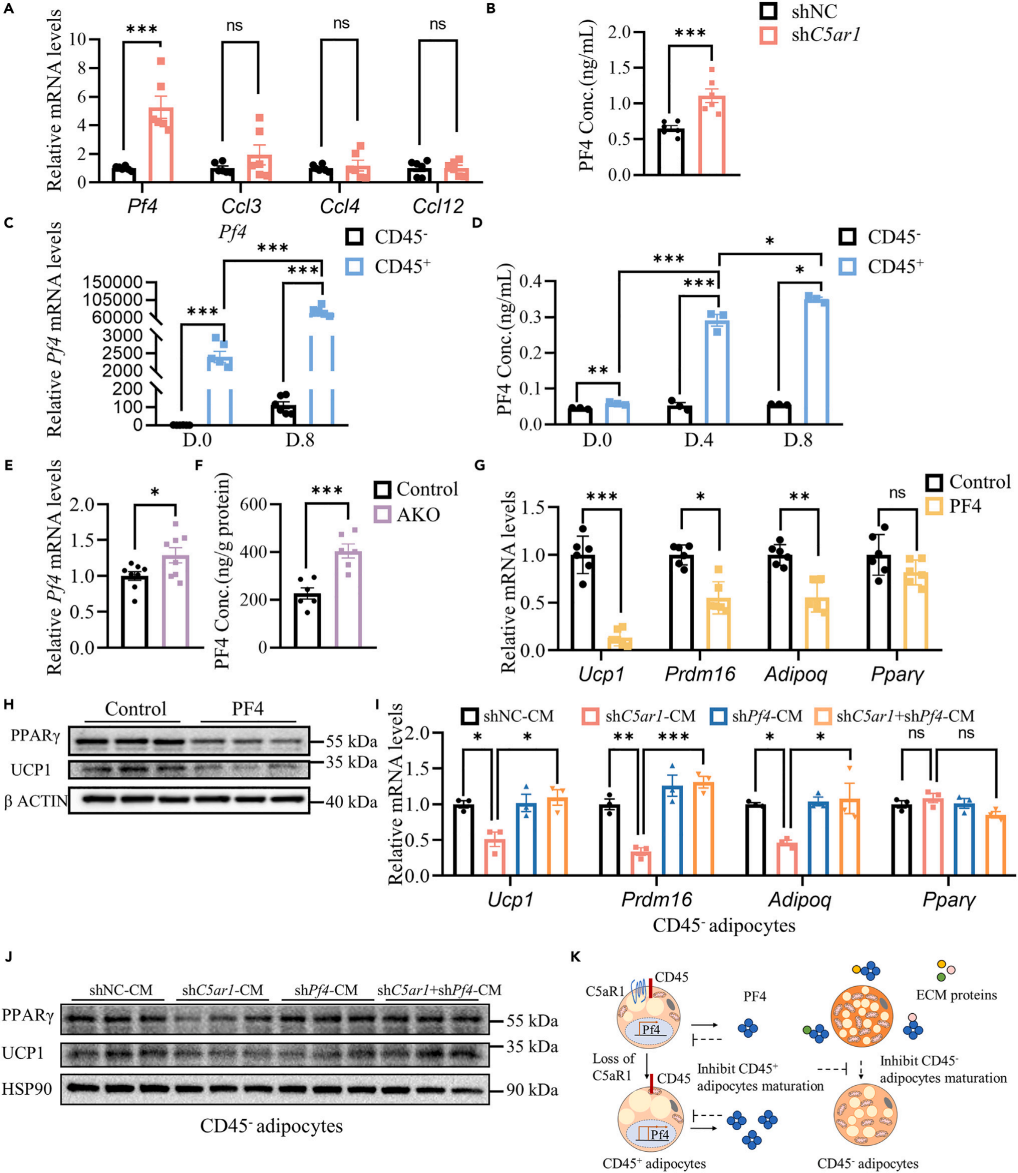
*C5ar1* knockdown in CD45^+^ brown adipocytes promote PF4 release to inhibit brown adipocyte maturation (A) Relative mRNA expression of the cytokines genes (*Pf4*, *Ccl3*, *Ccl4*, *Ccl12*) of *C5ar1* knockdown differentiated CD45^+^ adipocytes compared to the control group (*n* = 6). (B) The concentration of PF4 in the supernatant of *C5ar1* knockdown differentiated CD45^+^ adipocytes compared to the control group (*n* = 3). (C) *Pf4* mRNA expression in differentiated CD45^−^ and CD45^+^ adipocytes (*n* = 6). (D) Concentration of PF4 in the supernatant of differentiated CD45^−^ and CD45^+^ adipocytes (*n* = 3). (E) *Pf4* mRNA expression of BAT from Control and *C5ar1* AKO neonatal mice (*n* = 8). (F) Concentration of PF4 in BAT of Control and *C5ar1* AKO neonatal mice (*n* = 6). (G) Relative mRNA expression of the indicated genes of adipocyte differentiation of CD45^−^ ASCs cultured without or with 20 ng/mL PF4 (*n* = 6). (H) Immunoblotting for UCP1 and PPARγ of adipocyte differentiation of CD45^−^ ASCs cultured without or with 20 ng/mL PF4 (*n* = 3). (I) Relative mRNA expression of the indicated genes from adipocyte differentiation of CD45^−^ ASCs cultured in conditioned media from shNC, sh*C5ar1*, sh*Pf4* or sh*C5ar1*+*shPf4* CD45^+^ adipocytes (*n* = 3). (J) Immunoblotting for UCP1 and PPARγ of adipocyte differentiation of CD45^−^ ASCs cultured in conditioned media from shNC, sh*C5ar1*, sh*Pf4* or sh*C5ar1*+*shPf4* CD45^+^ adipocytes (*n* = 3). (K) Graphical abstract of this study: The loss of *C5ar1* in CD45^+^ adipocytes increased *Pf4* mRNA level and increased the secretion of PF4. PF4 inhibited the maturity and thermogenesis ability of both CD45^+^ and CD45^−^ adipocytes. Statistical significance was assessed by two-tailed Student’s t test (A–G) or one-way ANOVA (I). Data are represented as mean ± SEM ∗ ≤0.05, ∗∗ ≤0.01, ∗∗∗ ≤0.005.

Due to the small size of CD45^+^ adipocytes population in neonatal mice and with high cytokines mRNA level, we hypothesized that CD45^+^ adipocytes may regulate the thermogenesis of BAT by effecting the maturity of neighboring CD45^−^ adipocytes. When comparing RNA-seq data between CD45^+^ adipocytes and CD45^−^ adipocytes, CD45^+^ adipocytes showed higher mRNA levels of cytokines such as *Pf4*, *Ccl3*, *Ccl4*, and *Ccl12* (Figure 2G)*.* Thus, we tested the change in the expression of these cytokines after the elimination of *C5ar1* in CD45^+^ adipocytes using RT-qPCR. The result indicated that the *Pf4* mRNA was increased 5 times after *C5ar1* was knocked down in CD45^+^ adipocytes, which was the highest among 4 different cytokines in CD45^+^ adipocytes (Figure 4A). Consistently, the concentration of PF4 in the supernatant of *C5ar1* knockdown CD45^+^ adipocytes was higher than that of the control CD45^+^ adipocytes by measurement of enzyme-linked immunosorbent assay (ELISA) (Figure 4B). Furthermore, we found that *Pf4* mRNA expression increased rapidly (Figure 4C) and PF4 concentrations in the supernatant of CD45^+^ adipocytes were also enriched during the adipogenic differentiation of CD45^+^ adipocytes compared to CD45^−^ adipocytes (Figure 4D). The study revealed that the levels of *Pf4* mRNA in BAT of neonatal mice lacking the *C5ar1* were 1.5 times greater than those in control mice, while the PF4 protein levels in BAT of *C5ar1* AKO neonatal mice were approximately two times higher than in control mice (Figures 4E and 4F). Additionally, immunohistochemical analysis of PF4 in BAT samples from both control and C5ar1 AKO neonatal mice confirmed the elevated protein levels of PF4 in the C5ar1 AKO neonatal mice (Figure S4A). In general, PF4 could be secreted by CD45^+^ adipocytes and accumulate in the supernatant after the knock down of *C5ar1* in CD45^+^ adipocytes.

We further investigated the impact of PF4 on maturation of CD45^−^ adipocytes by planting CD45^−^ ASCs at confluency, followed by induction of adipogenic differentiation in the presence or absence of PF4. CD45^−^ adipocytes with PF4 showed approximately 80% lower expression of *Ucp1* and 50% lower expression of *Prdm16* compared to those without PF4, while the expression of the adipogenic marker *Pparγ* did not show significant change after treatment with PF4 (Figure 4G). Similarly to the removal of *C5ar1* in CD45^+^ adipocytes, UCP1 and PPARγ protein levels were decreased after the intervention of PF4 during CD45^−^ adipocyte differentiation (Figure 4H). These results demonstrated that PF4 could affect the maturation of CD45^−^ adipocytes. In order to assess the impact of PF4 on mature CD45^−^ adipocytes, PF4 was introduced to differentiated CD45^−^ adipocytes on day 6 of adipogenic differentiation for a duration of 48 h. Analysis through oil red O staining did not reveal any discernible differences between the PF4-treated group and the control group (Figure S4B). Furthermore, the relative mRNA expression levels of *Ucp1*, *Prdm16*, *Pparγ*, and *Adipoq* showed no significant variance between the two experimental groups (Figure S4C). Additionally, the protein levels of UCP1 and PPARγ remained unchanged following the administration of PF4 to CD45^−^ adipocytes (Figure S4D). Consequently, it was determined that PF4 did not exert any influence on the thermogenic capacity of CD45^−^ adipocytes.

In order to investigate the paracrine role of suppressing C5aR1 on CD45^+^ cells through PF4, experiments were conducted on CD45^−^ ASC using conditioned media from CD45^+^ adipocytes with knockdown of *C5ar1*, *Pf4*, or both *C5ar1* and *Pf4* via shRNA during adipocyte differentiation. The experimental design is depicted in Figure S4E. Initially, the efficacy of *C5ar1* and *Pf4* knockdown in CD45^+^ adipocytes was assessed, revealing reduced *C5ar1* mRNA expression in the sh*C5ar1* and sh*C5ar1*+*Pf4* groups, as well as decreased *Pf4* mRNA expression in the sh*C5ar1* and sh*C5ar1*+*Pf4* groups (Figure S4F). Subsequently, PF4 concentrations in the supernatant of CD45^+^ adipocytes with knockdown of shNC, sh*C5ar1*, sh*Pf4*, or shC5ar1+sh*Pf4* were measured. The results indicated that *C5ar1* knockdown led to an increase in PF4 levels in the supernatant of CD45^+^ adipocytes, which was rescued by simultaneous knockdown of both *C5ar1* and *Pf4* (Figure S4G). Then the conditioned media from CD45^+^ adipocytes as indicated treated were added to CD45^−^ ASCs during adipocyte differentiation and the sample were collected on differentiated day 8 (Figure S4H). Oil red O staining revealed reduced lipid accumulation in CD45^−^ adipocytes when exposed to conditioned media from sh*C5ar1* CD45^+^ adipocytes (Figure S4I). Conversely, exposure to conditioned media from sh*C5ar1*+sh*Pf4* CD45^+^ adipocytes resulted in increased lipid accumulation in CD45^−^ adipocytes (Figure S4J). This effect was further supported by a decrease in the relative mRNA levels of *Ucp1*, *Prdm16*, and *Adipoq* in CD45^−^ adipocytes treated with conditioned media from sh*C5ar1* CD45^+^ adipocytes, consistent with PF4 treatment (Figure 4G). Additionally, exposure to conditioned media from CD45^+^ adipocytes with knockdown of both *C5ar1* and *Pf4* led to an increase in the relative mRNA levels of *Ucp1*, *Prdm16*, and *Adipoq* (Figure 4I). Furthermore, when CD45^+^ adipocytes were cultured with conditioned media, the absence of *C5ar1* resulted in a reduction in the protein levels of PPARγ and UCP1 in CD45^−^ adipocytes. Conversely, when CD45^+^ adipocytes were cultured with conditioned media and both *C5ar1* and *Pf4* were eliminated, the decrease in protein levels was reversed (Figure 4J and S4K). These findings suggest that the knockdown of *C5ar1* in CD45^+^ brown adipocytes enhance the release of PF4, which in turn inhibits the maturation and thermogenic capacity of CD45^−^ adipocytes.

It is uncertain whether the inhibition of *Pf4* in CD45^+^ adipocytes can enhance thermogenic activity. An experiment was conducted to silence *Pf4* in CD45^+^ adipocytes using shRNA, and subsequently assess the levels of lipid accumulation in cells, as well as the expression of thermogenesis-related genes and proteins. The reduction in Pf4 expression was confirmed at both the mRNA and protein levels in these cells (Figures S5B and S5C). Following the knock-down of *PF4*, oil red O staining revealed elevated lipid accumulation in CD45^+^ adipocytes (Figure S5A). Concurrently, the expression of thermogenesis-related genes such as *Ucp1* and *Prdm16* was notably upregulated in these cells in the absence of *Pf4* (Figure S5B). Furthermore, the depletion of *Pf4* resulted in a significant increase in the protein levels of PPARγ and UCP1 in these cells (Figure S5D). In summary, the inhibition of *Pf4* in CD45^+^ adipocytes appears to enhance thermogenic activity.

These findings strongly indicated that *C5ar1* knockdown in CD45^+^ brown adipocytes promote the release of PF4 and PF4 inhibited the maturity and thermogenesis ability of brown adipocytes (Figure 4K).

## Discussion

Several studies have attempted to determine the gene profiles of adipose progenitor cell (APC) populations in the SVF of adipose tissue by means of scRNA-seq analysis.^18^^,^^19^^,^^20^^,^^21^ It has been confirmed that all APCs express cell surface markers including *Cd9*, *Ly6a(Sca1)*, *Pdgfrα*, *Cd34*, which can be reliably used to identify and recruit these cells.^12^^,^^22^^,^^23^ Three populations of ASCs have been identified in mice BAT and the percentages of each population change during cold simulation.^13^ However, the enrichment of preadipocyte populations, as well as their roles in the regulation of thermogenesis, is still unknown in BATs of neonatal mice, which require further exploration. In this study, we focus on clarifying a distinct group of brown adipocytes in the BAT of neonatal mice, trying to figure out the characteristics and regulatory mechanisms of this subpopulation. According to the single-cell RNA-seq and FACS analysis of SVF from BAT of neonatal mice, we surprisingly identified a specific ASC population except for five known populations of ASCs (Figure 1C). They expressed both the adipogenic markers CD29, SCA1 and the immune linage marker CD45, so we termed these cells CD45^+^ ASCs (Figure 1E). When separated neonatal mice SVF by FACS, these cells adherent to cell culture plates and could be cultured and proliferated to their confluency in several days (Figure S1F). The CD45^+^ adipose progenitor cells were isolated and morphologically shown *in vitro.*^24^ Moreover, the confluent CD45^+^ ASCs could be differentiated into mature brown adipocytes with a larger cell size than other ASCs and immune cells (Figure 2A). Based on these results, we believe that these CD45^+^ ASCs belong to a unique subgroup of ASCs without losing their linage markers during differentiation. Several previous studies have reported the possibility that myeloid-derived stem cells could be recruited to adipose tissues during cold stimulation; however, they lost linage markers during their residence in adipose tissues.^24^ CD45^+^ adipocytes were different from classical CD45^−^ adipocytes, which have high thermogenic ability and low levels of cytokines. In addition to increased cytokine expression, CD45^+^ adipocytes also showed higher level of fatty acid metabolism and inflammation response (Figure 2G). The mRNA levels of immune response genes were up-regulated during CD45^+^ adipocyte differentiation, which may participate in the regulation of BAT maturation in the postnatal stage.

C5aR1 belongs to the family of GPCRs^25^ and is known to have pleiotropic functions such as maintaining tissue homeostasis, playing roles in innate and adaptive immune networks.^26^^,^^27^^,^^28^ It is mainly expressed in most immune cells, however, the expression of C5aR1 in tissue cells remained controversial.^29^^,^^30^^,^^31^ Recent studies have shown that loss of *C5ar1* in immune cells could affect the secretion of cytokines,^32^ impede tumorigenesis,^33^ and inhibit cell migration.^34^ In this study, we found that the expression of *C5ar1* was higher in CD45^+^ adipocytes than in CD45^−^ adipocytes and increased during adipogenic differentiation (Figure 2H). *C5ar1* knockdown in CD45^+^ adipocytes caused a decrease in thermogenic gene expression and a decrease in UCP1 and PPARγ protein levels (Figure 3D). Furthermore, adipocyte-specific deletion of *C5ar1* in neonatal mice resulted in immaturity of brown adipocytes and therefore a reduction in cold tolerance (Figure 3G). We also observed the level of *C5ar1* throughout the life cycle of *C5ar1* AKO mice, the level of *C5ar1* only declined in neonatal stage (Figure S3I) is consistent with previous findings that these CD45^+^ adipocytes only appeared in neonatal mice and disappeared before weaning (the week four after delivery).^13^ BAT of *C5ar1* AKO neonatal mice also contained larger lipid drops in cells and a lower protein level of UCP1 than that of the control mice (Figure 3J). Thus, it was suggested that CD45^+^ adipocytes are unique in the BAT of neonatal mice and that C5aR1 on their membrane is prominent for its regulation on thermogenesis.

Notably, extremely high expression of *Pf4* was observed in CD45^+^ adipocytes as well as in the supernatant of CD45^+^ adipocytes with *C5ar1* deficiency, suggesting PF4 release from these cells after *C5ar1* removal (Figures 4A and 4B). PF4 was a special chemokine with multiple biological functions, such as hematopoietic and angiogenic inhibition,^35^^,^^36^^,^^37^ leukocyte activation and recruitment,^38^ cell proliferation and differentiation.^37^^,^^39^ PF4 plays its role through three main ways: interaction with its receptors, glycosaminoglycans (GAGs)^40^ and formation heterodimers with other chemokines such as Cxcl12.^41^ Moreover, during CD45^+^ adipocyte differentiation, induction of *Pf4* expression was detected at both mRNA level in cells and protein level in cell culture supernatant (Figures 4C and 4D). Treatment with recombinant protein PF4 during CD45^−^ adipocyte differentiation led to a downregulation of thermogenic genes, such as *Ucp1* and *Prdm16.* However, no change in mRNA expression but a decrease in PPARγ protein expression was observed in these cells after PF4 treatment (Figure 4G). We also found that PF4 could not affect the thermogenesis ability of differentiated CD45^−^ adipocytes (Figures S4B–S4D). Knockdown *Pf4* in CD45^+^ adipocytes could increase cells thermogenic activity (Figure S5). These results indicated that PF4 could be released from CD45^+^ adipocytes and influence the maturation of brown adipocytes and thermogenesis ability.

In conclusion, our study revealed the existence of a population of brown adipocytes derived from CD45^+^ ASCs in neonatal mice. They participated in the regulation of thermogenesis in newborns through C5aR1. A knockdown of *C5ar1* in CD45^+^ adipocytes led to induced secretion of PF4, which suppressed the maturity of neighbor adipocytes. Our findings unveiled the mechanism of BAT-mediated thermogenesis during the neonatal stage, which replenished the knowledge on neonatal adaptation to ambient temperature and may provide possibilities to promote BAT thermogenesis in adults through these targets.

### Limitations of the study

While we sorted out cultured and differentiated CD45^+^ ASCs *in vitro*, the roles of CD45^+^ adipocytes *in vivo* need further exploration through their loss or gain experiments. Our study, for the first time, presented the effects of PF4 on the maturation of brown preadipocytes. Other cytokines, that are found to be expressed higher in CD45^+^ adipocytes compared to CD45^−^ adipocytes, might also affect the thermogenesis of neonatal mice and require further investigation.

## Resource availability

### Lead contact

Further information and requests for resources and reagents should be directed to and will be fulfilled by the lead contact, Yong Chen (tj.y.chen@vip.163.com).

### Materials availability

This study did not generate new unique reagents.

### Data and code availability

• Single-cell RNA-seq data generated during this study have been deposited at the National Center for Biotechnology Information Sequence Read Archive database and are publicly available as of the date of publication. RNA-seq data generated in this study have been deposited at the National Center for Biotechnology Information Sequence Read Archive data and are publicly available as of the date of publication. Accession numbers are listed in the key resources table. All data reported in this paper will be shared by the lead contact upon request.
• This paper does not report original code.
• Any additional information required to reanalyze the data reported in this paper is available from the lead contact upon request.

## Acknowledgments

This work was supported by the National Natural Science Foundation of China & National Key Research and Development program of China (82070859 to Y.C. & 2023YFC2706300 to X.P.L.). We also thank the other grants from the 10.13039/501100001809National Natural Science Foundation of China (82350610277, 82270910 to Y.C.), and 10.13039/501100012166National Key Research and Development Program of China (2022YFA0806100 to Y.C.). We appreciate for the help of single cell analysis by Xi Wang, and Xia Li from Wuhan Biobank Co.,Ltd.

## Author contributions

H.-Y.W., X.-P.L., and Y.C. conceived the project. H.-Y.W. designed and carried out experiments and analysis the data. H.-Y.W. and X.-M.P. performed scRNA-seq analyses. X.-M.P., M.Y., and X.Y. assisted with the animal and cellular experiments. Y.W. offered technical assistance. D.Z. assisted with studies in AKO mice. Y.C., X.-P.L., and Q.N. guided research. H.-Y.W. and Y.C. wrote the manuscript, Y.C. and X.-P.L. edited the manuscript. All authors read and approved the manuscript.

## Declaration of interests

The authors declare no competing interests.

## STAR★Methods

### Key resources table

**Table:** 

REAGENT or RESOURCE	SOURCE	IDENTIFIER
**Antibodies**		

Brilliant Violet 421™ anti-mouse CD45 Antibody	Biolegend	RRID: AB_2562559
PE/Cyanine7 anti-mouse Ly-6A/E (Sca-1) Antibody	Biolegend	RRID: AB_493596
APC anti-mouse/rat CD29 Antibody	Biolegend	RRID: AB_492833
PE anti-mouse CD88 (C5aR) Antibody	Biolegend	RRID: AB_2243735
Anti-UCP1 antibody	Sigma-Aldrich	Cat#U6382; RRID: AB_261838
Anti-PPARγ antibody	Cell Signaling Technology	Cat#2435T
Anti-C5aR1 antibody	Abcam	Cat#59390; RRID: AB_2243718
β-Actin Rabbit monoclonal Antibody	Abclonal	Cat#AC038
Hsp90α/β Rabbit monoclonal Antibody	Abclonal	Cat#A5027; RRID: AB_2863419
Alpha Tubulin Monoclonal antibody	Proteintech	Cat# 66031-1-Ig; RRID: AB_2883483
Anti-CD29 Rabbit monoclonal Antibody	Abclonal	Cat#A23497
CD45 monoclonal Antibody	Proteintech	Cat# 60287-1-Ig; RRID: AB_2881404
HRP Goat Anti-Rabbit IgG (H + L)	Abclonal	Cat#AS014; RRID: AB_2769854
HRP Goat Anti-Mouse IgG (H + L)	Abclonal	Cat#AS003; RRID: AB_2769851

**Chemicals, peptides, and recombinant proteins**		

Recombinant mouse PF4 protein	MCE	Cat# HY-P71885
DMEM/F12	Hyclone	Cat#SH30023.01
FBS	Cellmax	Cat#SA211.02
Collagenase II	Sigma	Cat#C6885
Insulin	Sigma	Cat#bs901
T3 (3,30,5-Triiodo-L-thyronine sodium salt)	Sigma	Cat#T6397
IBMX (3-Isobutyl-1-methylxanthine)	Sigma	Cat#I5879
Dexamethasone	Sigma	Cat#D4902
Indomethacin	Sigma	Cat#I7378
RNAiso Plus	TaKaRa	Cat#9108

**Critical commercial assays**		

Zombie Aqua™ Fixable Viability Kit	Biolegend	Cat#423101
Mouse PF4/CXCL4 ELISA Kit	Boster	Cat#EK0727
Hifair® Ⅲ 1st Strand cDNA Synthesis SuperMix	Yeasen	Cat#11141ES60
Hieff UNICON® qPCR SYBR Green Master Mix	Yeasen	Cat#11198ES08

**Deposited data**		

Single-cell RNA sequence data	This paper	SRA:PRJNA1166873.
RNA sequence data for CD45^−^ and CD45^+^ adipocytes	This paper	SRA:PRJNA1166695
RNA sequence data for neonatal mice	This paper	SRA:PRJNA1166696

**Experimental models: Organisms/strains**		

*Adipoq-Cre*: B6; FVB-Tg (Adipoq-cre)1Evdr/J	The Jackson Laboratory Stock	No: 010803
Mouse: *C5ar1*^*flox/flox*^	Shanghai Model Organism Center	Cat#NM-CKO-200282

**Oligonucleotides**		

Primers for RT-qPCR	Table S1	

**Software and algorithms**		

FLIR Tools	FLIR	https://www.flir.com/products/support
Graphpad Prism 8	GraphPad Software	N/A
FlowJo	v10	https://www.flowjo.com
Bowtie2	v2.2.5	http://bowtiebio.sourceforge.net/bowtie2/
RSEM	v1.2.8	http://deweylab.biostat.wisc.edu/RSEM
DEseq2		https://bioconductor.org/packages/release/bioc/html/DESeq2.html
Excel	Microsoft	
Cell Ranger Single Cell Software Suite	V3	https://www.10xgenomics.com
Seurat	V4.2.3	https://satijalab.org/seurat/

### Experimental model and study participant details

#### Animals

All mice were on the C57BL/6J genetic background. C5ar1 AKO mice (C5ar1^flox/flox^; AdipoqCre^+^) and Control (C5ar1^flox/flox^; AdipoqCre^−^) mice were generated by breeding C5ar1^flox/flox^ mice (generated by Shanghai Model Organisms Center) with Adiponectin-Cre (Jackson Laboratories) mice to produce C5ar1 AKO mice. The strategy for the generation of mice was described in the genomic structure (Figure 3E). Neonatal mice (at postnatal day1) were used for the studies unless otherwise stated. Mice were housed in a 12-h light/12-h dark cycle of food and water *ad libitum* and in a pathogen-free barrier facility at 22.0–26.0 °C. All animal experiments were approved by Tongji Hospital Laboratory Animal Welfare & Ethics Committee, affiliated with Huazhong University of Science & Technology (Approval No:TJ-IRB202104015). Mice were euthanized by carbon dioxide (CO2) asphyxiation inhalation and cervical dislocation was performed as secondary euthanasia procedure, and then the tissues were isolated.

### Method details

#### BAT-SVF cells isolation, culture, and differentiation

BAT depots from neonatal mice (at postnatal day1) were collected, pooled and cut up in 10 mL of digestion buffer (Hank’s buffer solution with 1 mg/mL Collagenase II, 4%BSA, and 1% Pen-Strep). After shaking in 37 °C at 100 rpm for 30 min, digested tissues were neutralized with PBS solution with 1% FBS and filtered through 70μm filters. Centrifuged at 300g for 5 min and the pellets were resuspended in red blood cell lysis buffer and put on ice for 2 min to remove red blood cells. The cell suspension was neutralized with same volume of PBS plus 10% FBS and removed after a 300g centrifugation for 5 min. The pelleted cells were resuspended in PBS. Cells were then stained with desired antibodies, and subjected to FACS. Subsequently, CD45^−^ (CD45^−^, SCA1^+^, CD29^+^) and CD45^+^ (CD45^+^, SCA1^+^, CD29^+^) ASCs were isolated using FACS and seeded onto collagen-coated plates. To immortalization the SVF, after 48h, adherent SVF cells were infected with lentivirus expressing CMV-SV40gp6-WPRE. When confluent, infected SVF cells were passed into T75 culture bottle cultured with DMEM/F12 (10% FBS, 1% Pen-Strep) and labeled as passage 1 (P1). Cells with less than 5 passages were used. At 100% confluency, cells were induced to differentiate for 2 days with an adipogenic cocktail (0.5mM IBMX, 2μg/ml dexamethasone, 850nM insulin, 1nM T3, 125μM indomethacin) in DMEM/F12 medium containing 10% FBS. Two days after induction, cells were refed every 48 h with adipocyte culture medium containing 1nM T3, and 850nM insulin. Cells were fully differentiated by day8 after induction. Lipid droplets were visualized by Oil red O staining.

#### Single-cell RNA sequencing and analysis

SVF were isolated from BAT of neonatal mice (at postnatal day1) at room temperature. We performed sc-RNA seq three times. For each scRNA-seq, 6–7 mice from each age group were used. Red blood cells were removed from SVF fraction with a red blood cell lysis, and live cells were sorted by AO positive cells which were stained with AO/PI dye before FACS. The scRNA-seq experiment was carried out by Wuhan biobank. The sorted single-cell suspension, 10x 3′ barcoded gel beads, and oil were loaded into Chromium Single Cell G Chip to capture single cells in nanoliter-scale oil droplets by Chromium Controller and to generate Gel Bead-In-EMulsions (GEMs) and single-cell RNA-seq libraries were obtained following the 10x Genomics protocol using reagents included in the Chromium Single Cell 3′ v3.1 Reagent Kit. The resulting libraries were sequenced on an Illumina NovaSeq 6000 instrument.

10X Genomics Cell Ranger (v3) was used to perform sample quality control filtering, comparison, quantification, identification and recovery of cells on the original data. The number of tags per gene was calculated, and cells were recognized following the requirement, which resulted in approximately 8000 cells, with 40,000–80,000 mean reads/cell and 3000 median genes detected per cell. Seurat (v3.1.5) was used to trim dataset (nFeature_RNA >100, nFeature_RNA <6380, percent.mt < 10), normalize data (LogNormalize). Highly variable genes were used for principal component analysis, followed by clustering in PCA space using a graph-based clustering approach (dims = 1:10, resolution = 0.3). UMAP and tSNE were then used for 2D visualization of the resulting cluster. Marker genes were identified using the FindAllMarkers function (only.pos = TRUE, min.pct = 0.25, logfc.threshold = 0.25). Cluster analysis was performed on differentially expressed genes, and top 200–400 genes expressed in each cluster was used for cell type identification. Differentially expressed genes (DEGs) were used to identify each cluster, and two-way ANOVA with Bonferroni test was utilized for multiple comparisons to calculate statistical significance of upregulated genes and pathways. We used known cell-type specific markers curated from literature and previous studies in SVF.

#### Oil red O staining

Cells were washed twice with PBS and fixed with 4% paraformaldehyde and washed twice with PBS, then stained with a filtered Oil Red O working solution (Sigma-Aldrich, O0625) for 30 min at room temperature. Then cells were got white light photography.

#### Gene expression analysis

Total RNA was extracted from tissue or cells using RNAiso Plus (TaKaRa, 9108), and 1mg total RNA was reverse transcribed to cDNA using the Hifair Ⅲ 1st Strand cDNA Synthesis SuperMix for qPCR (Yeasen, 11141ES60) according to the manufacturers’ instructions. Quantitative real-time PCR was performed using the Hieff UNICON qPCR SYBR Green Master Mix (Yeasen, 11198ES08) on the CFX96 connection System (BioRad). The expression levels of genes were calculated using the delta Ct method, after normalization to 36B4 expression (for cells samples) or Hprt (for tissue samples). Sequences of primers used for real-time PCR are listed in Table S1. Experiments were repeated three times and showed the mean value.

#### RNA-sequencing analysis

RNA quality was examined with Fragment Analyzer. High quality RNA was used to construct library and Illumina NovaSeq6000 was used to perform RNA-seq (BGI Genomics, China). Briefly, library was prepared at BGI and sequencing was performed using the DNBSEQ T7 platform Processed RNA-seq data were filtered by removing genes with low read counts. We used Bowtie2 matched clean reads to reference gene sequences, and then used RSEM to calculate gene expression levels of each sample. DESeq2 were used to determine differential genes with the following parameters: adjusted *p*-value ≤ 0.05. KEGG pathway analysis was performed by KEGG profile. *p*-value <0.05 was considered as significantly enrichment.

#### Immunoblotting and antibodies

Cells and tissues were lysed in RIPA lysis buffer (Beyotime, P0013B) containing PMSF and protease inhibitors. Total protein lysates were boiled for 5 min and loaded on a 10% SDS-PAGE. Subsequently, separated proteins were transferred onto PVDF membranes. PVDF membrane blots were blocked in 5% skimmed milk for 1h at room temperature, washed in Tris-buffered saline with Tween 20 (TBST) and incubated overnight at 4°C with rabbit anti-UCP1 (1:1000, Sigma, U6382), anti-C5aR1 (1:500, Abcam, ab59390), anti-PPARγ (1:1000, CST, 2435), anti-β-ACTIN (1:5000, ABclonal, AC038), anti-HSP90 (1:1000, ABclonal, A5027), Mouse anti-α-TUBLIN (1:200000, Proteintech, 66031-1-Ig). Anti-rabbit IgG (ABclonal, AS014) was used as the second antibody for UCP1, C5aR1, PPARγ, β-ACTIN and HSP90. Anti-mouse IgG (ABclonal, AS003) was used as the second antibody for α-TUBLIN.

#### Flow cytometry

SVF cells were incubated with Zombie Aqua Fixable Viability Kit for 10min and then indicated antibody for 15min in the dark, washed, centrifuged at 300g for 5 min, resuspended in PBS and passed through a 40μm filter prior to FACS analysis. FACS were performed on Beckman MoFlo XDP Cell Sorter. Cells were initially chosen based on forward scatter (FFC) and side scatter (SSC) and then chosen the live cells. Cell population (%) was calculated as frequency of parent. FlowJo software (version 10.6.2) was used for data analysis.

#### Histological analysis and immunohistochemistry

For hematoxylin and eosin (H&E) staining, tissues of mice were fixed in 4% paraformaldehyde (PFA) overnight, followed by dehydration. After the dehydration procedure, tissues were embedded in paraffin, then sectioned at a thickness of 2–3 μm and stained with H&E following the standard protocol. Images were acquired using a Pannoramic SCAN (3DHISTECH). Adipocyte sizes were quantified by ImageJ.

For immunostaining, paraffin-embedded tissue sections were deparaffinized in xylene and subsequently rehydrated. After deparaffination, samples were blocked hydroperoxides by 3% H2O2 solution for 25 min and washed with PBS three times and then blocked in 5% BSA for 30 min at room temperature. After washing in PBS, slides were incubated with rabbit anti-UCP1(1:200, ab10983, Abcam), rabbit anti-PF4 (1:200, Boster, PB1063) overnight at 4°C and then incubated with the secondary antibody for 1 h. The nucleus was stained by hematoxylin for 5 min. Images were acquired using a Pannoramic SCAN (3DHISTECH).

#### Skin temperature and cold tolerance

Neonatal mice (at postnatal day 1) were exposed from 30°C to 22°C for 5min to test their cold tolerance. Their back skin temperature (focused on the area of interscapular BAT) was monitored and pictures were taken with an FLIR-C3 thermal camera and measured with the FLIR tool.

#### ELISA

The supernatant was collected by centrifugation at 14,000 g at 4°C and analyzed by ELISA using a mouse PF4/CXCL4 ELESA Kit (Boster, Ek0727) following the manufacturer’s instructions.

### Quantification and statistical analysis

#### Statistical analysis

Statistical analyses were performed with Prism software version 8.0.1 (GraphPad Software). Statistical analyses were performed using two tailed t-test or one-way ANOVA. The error bars represent standard deviation (SEM). Data are expressed as mean ± SEM and *p*-value<0.05 was considered statistically significant. Number of mice or replicates used in each experiment was indicated in figure legends. Experiments were repeated at least three times.
