# Supplementary material for: C5aR1-positive adipocytes mediate non-shivering thermogenesis in neonatal mice
Source: iScience. 2024 Oct 28;27(12):111261. doi: 10.1016/j.isci.2024.111261 (PMC11700647; doi:10.1016/j.isci.2024.111261)
Supplement: Document S1. Figures S1–S5 and Table S1 [file mmc1.pdf]

iScience, Volume *27*

Supplemental information

C5aR1-positive adipocytes mediate non-shivering thermogenesis in neonatal mice

Huan-Yu Wang (汪欢玉), Xue-Min Peng (彭雪敏), Min Yang (杨敏), Ying Weng (翁莹), Xi Yang (杨希), Di Zhan (詹迪), Qin Ning (宁琴), Xiao-Ping Luo (罗小平), and Yong Chen (陈勇)

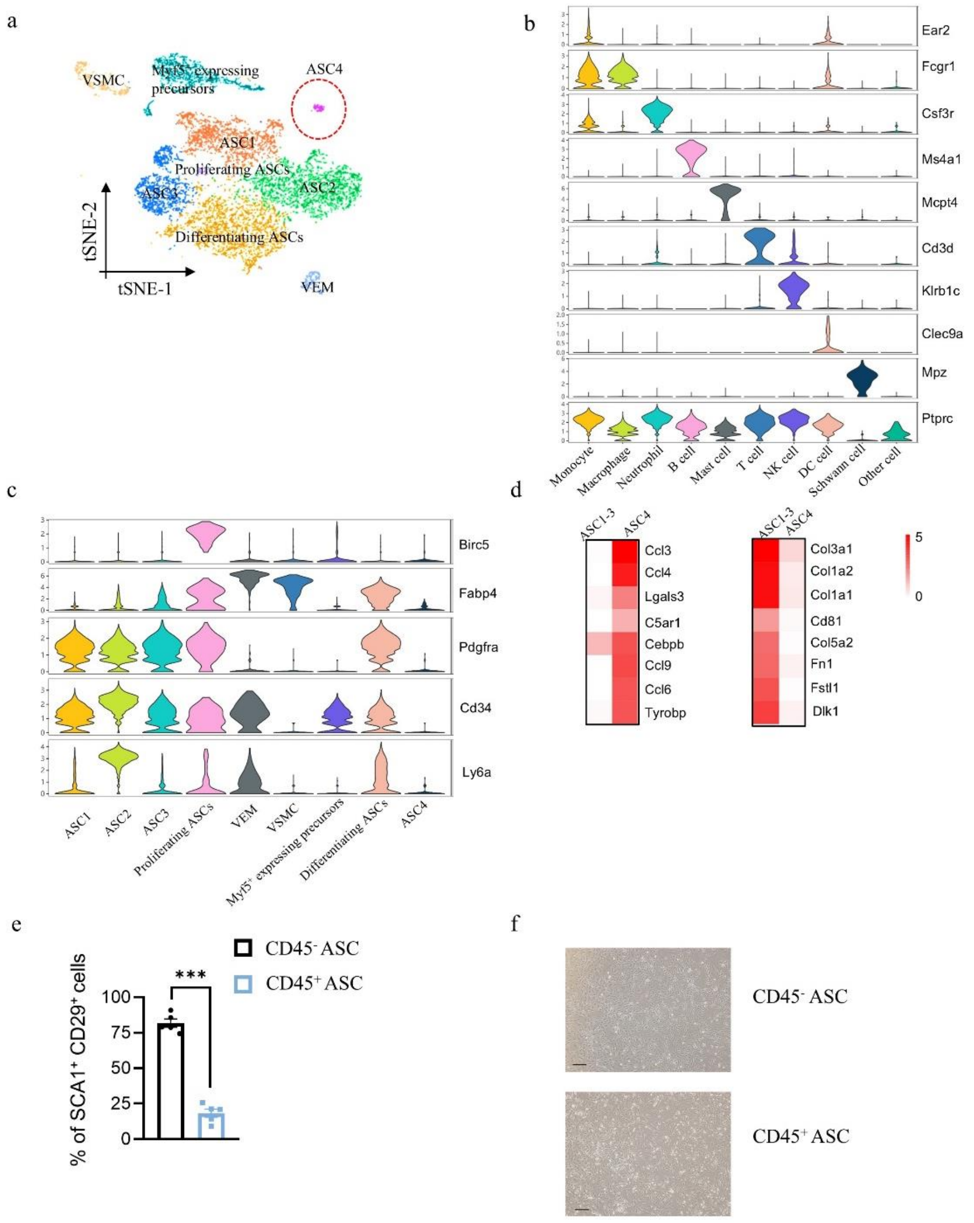

Figure S1. Identification of the CD45$^+$ fibroblasts population in BAT of neonatal mice, related to Figure 1

(a) tSNE plot revealing SVF populations of non- immune cells isolated from BAT from neonatal mice.

(b) Normalized gene expression value as violin plots of selected cluster-specific genes from immune cells from neonatal mice.

(c) Normalized gene expression value as violin plots of selected cluster-specific genes from non-immune cells of neonatal mice.

(d) Heatmap of differentially expressed genes down-regulated and up-regulated in ASC4 compared to ASC1-3.

(e) Percentage of CD45$^-$ and CD45$^+$ ASCs in BAT SVF of neonatal mice (n = 5).

(f) In vitro cell culture of CD45$^-$ and CD45$^+$ ASCs reaching confluence. Sale bar = 100μm.

Data are represented as mean ± SEM * ≤ 0.05, ** ≤ 0.01, *** ≤ 0.005.

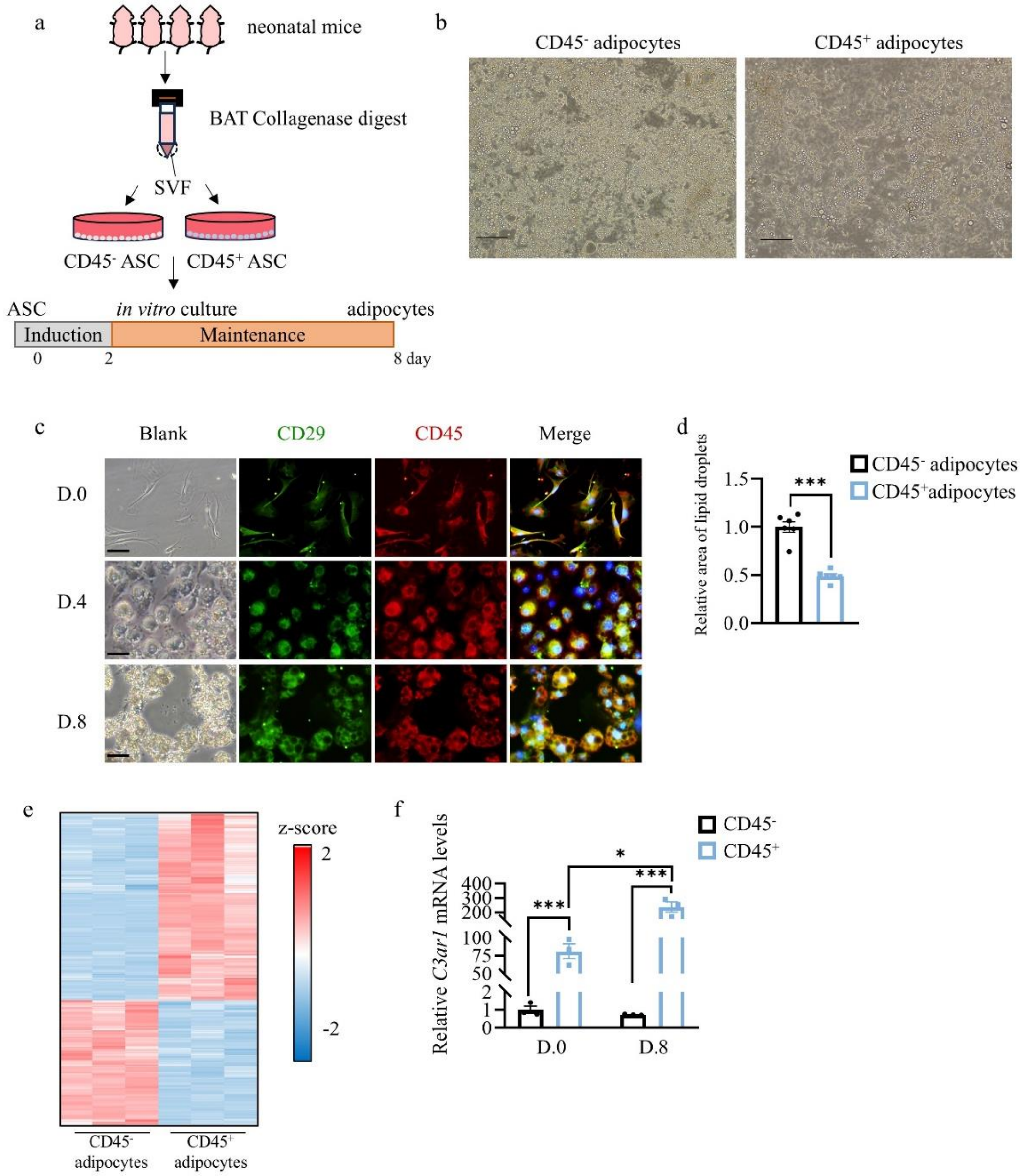

Figure S2. In vitro differentiated CD45$^+$ adipocytes were distinct from classical brown adipocytes, related to Figure 2

(a) Schematic illustration of experiments in culture cell studies. CD45$^-$ ASCs and CD45$^+$ ASCs were isolated from the BAT of neonatal mice and differentiated for 8 days under an adipogenic condition.

(b) Brightfield image of unstained CD45$^-$ and CD45$^+$ adipocytes differentiated for 8 days in adipogenic conditions. Scale bar = 100 µm.

(c) Co-immunofluorescence staining during adipogenic differentiated of CD45$^+$ ASCs for CD45, CD29 on day 0, day 4 and day 8. Scale bars = 10µm.

(d) Standard quantification of lipid accumulation via the amount of Oil red O staining of adipogenic differentiation of isolated CD45$^-$ and CD45$^+$ ASCs *in vitro*. (n = 6)

(e) RNA-seq of CD45$^-$ and CD45$^+$ adipocytes. Heat map of the global gene expression profile. (n = 3).

(f) Expression of *C3ar1* mRNA in CD45$^-$ and CD45$^+$ ASCs and adipogenic differentiated CD45$^-$ and CD45$^+$ adipocytes.

Data are represented as mean ± SEM * ≤ 0.05, ** ≤ 0.01, *** ≤ 0.005.

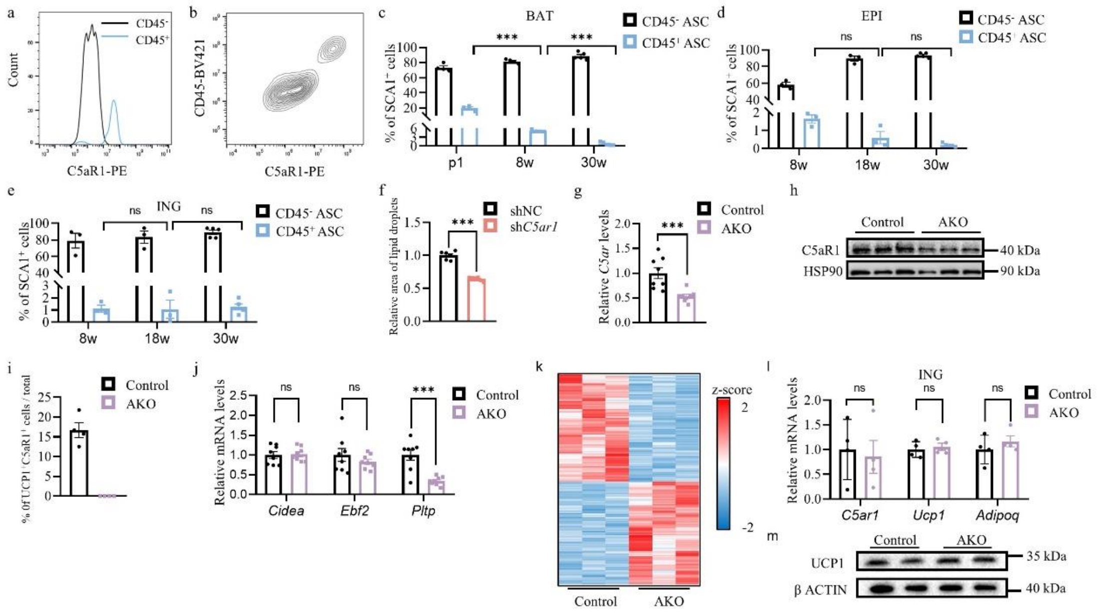

Figure S3. Loss of the *C5ar1* gene in adipocytes during the perinatal period decreased the thermogenesis ability of neonatal mice, related to Figure 3

(a) The expression level of C5aR1 of CD45$^-$ and CD45$^+$ preadipocytes from SCA1$^+$, CD29$^+$ cells of SVF of BAT from neonatal mice.

(b) CD45 and C5aR1 expression levels in SCA1$^+$CD29$^+$ cells in the SVF of BAT of neonatal mice. Note that all the CD45$^+$SCA1$^+$CD29$^+$ cells are marked by C5aR1.

(c) Percentage of CD45$^+$CD88$^+$ ASC and CD45$^-$ ASC in Sca1$^+$ cells of brown adipose tissue (BAT) of neonatal, 8w and 30w mice (n = 3).

(d) Percentage of CD45$^+$CD88$^+$ ASC and CD45$^-$ ASC in Sca1$^+$ cells of epididymal visceral adipose tissue (EPI) of 8w, 18w and 30w mice (n = 3).

(e) Percentage of CD45$^+$CD88$^+$ ASC and CD45$^-$ ASC in Sca1$^+$ cells of inguinal adipose tissue (ING) of 8w, 18w and 30w mice (n = 3)

(f) Standard quantification of lipid accumulation via the amount of Oil red O staining of Oil red O staining of shNC and sh*C5ar1* CD45$^+$ adipocytes *in vitro* (n = 6).

(g) The expression level of *C5ar1* in mRNA in the BAT of neonatal mice was significantly suppressed by the depletion of C5aR1 in adipocytes (n = 8 mice per group).

(h) The expression level of C5aR1 in protein in BAT of neonatal mice was significantly suppressed by depletion of *C5ar1* in adipocytes (n = 3 mice per group).

(i) Percentage of C5aR1$^+$UCP1$^+$ cells in the BAT of the control and *C5ar1* AKO neonatal mice (n = 4). Related to Figure 3F.

(j) The relative mRNA expression level of indicated genes of BAT from control and *C5ar1* AKO neonatal mice (n = 8).

(k) RNA-seq of BAT of the control and *C5ar1* AKO neonatal mice. Heat map of the global gene expression profile. (n = 3).

(l) The expression level of *C5ar1, Ucp1* and *Adipoq* in mRNA in the inguinal adipose tissue of Control and *C5ar1* AKO 8w male mice (n = 4 mice per group).

(m) Immunoblotting for UCP1 of inguinal adipose tissue from control and *C5ar1* AKO 8w male mice (n = 2).

Data are represented as mean ± SEM * ≤ 0.05, ** ≤ 0.01, *** ≤ 0.005.

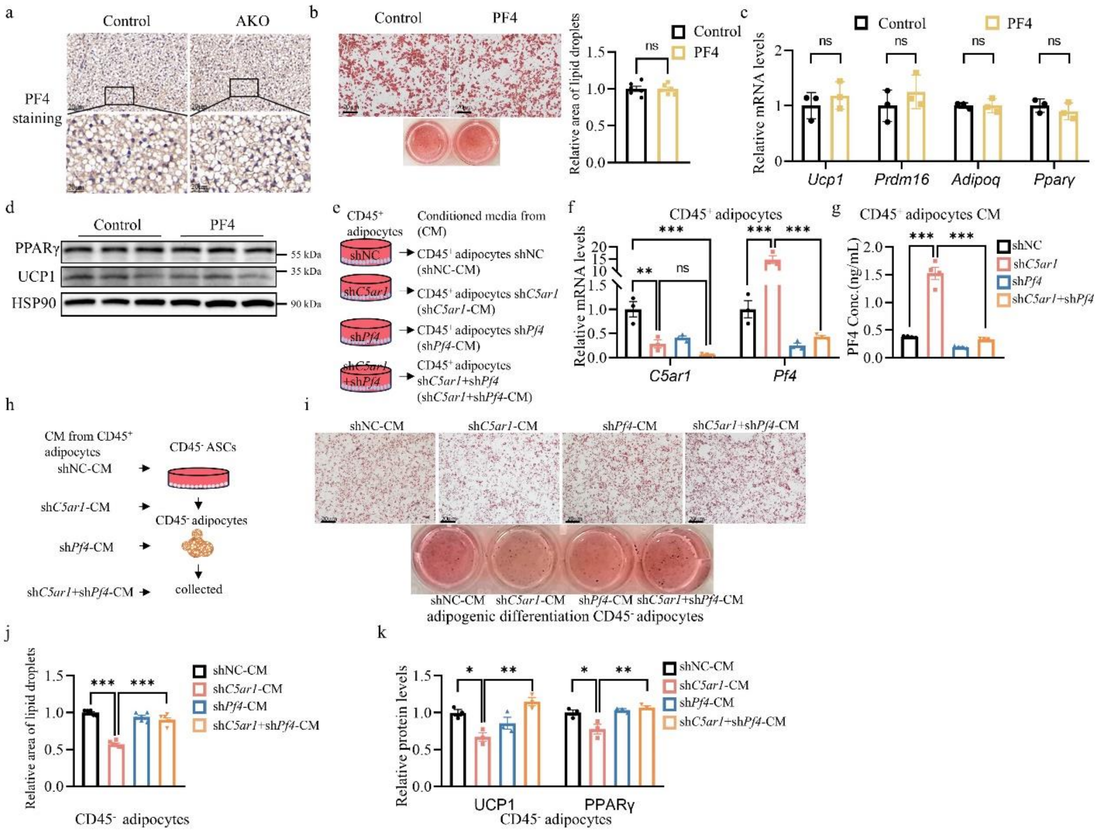

Figure S4. C5aR1 knockdown in CD45⁺ brown adipocytes promoted the release of PF4 to inhibit the maturation of brown adipocytes, related to Figure 4.

(a) Immunohistochemical staining with PF4 of BAT from Control and *C5ar1* AKO neonatal mice.
(b) Oil red O staining of CD45⁻ brown adipocytes after stimulation with 20 ng / mL PF4 for 48h at adipogenic differentiated day 6 and quantification of lipid accumulation (n = 6). Scale bars = 20μm
(c) Relative mRNA expression of the indicated genes of CD45⁻ brown adipocytes after stimulation with 20 ng / mL PF4 for 48h at adipogenic differentiated day 6 (n = 3).
(d) Immunoblotting for UCP1 and PPARγ of CD45⁻ brown adipocytes after stimulation with 20 ng / mL PF4 for 48h at adipogenic differentiated day 6 (n = 3).
(e) Schematic illustration of experiments in culture cell studies. The knockdown of *C5ar1* or *Pf4* in CD45⁺ ASCs was performed using shRNA, then the treated CD45⁺ ASCs were differentiated into mature adipocytes and the conditioned media were collected on differentiated day 8.
(f) The mRNA expression of *C5ar1* and *Pf4* in shNC, sh*C5ar1*, sh*Pf4* or sh*C5ar1+shPf4* CD45⁺ adipocytes (n =3).
(g) Concentration of PF4 in the supernatant of shNC, sh*C5ar1*, sh*Pf4* or sh*C5ar1+shPf4* CD45⁺ adipocytes (n =4).
(h) The conditioned media from CD45⁺ adipocytes as indicated treated were added to CD45⁻ ASCs during adipocyte differentiation and the sample were collected on differentiated day 8.
(i) Oil red O staining of CD45⁻ brown adipocytes cultured in conditioned media from shNC, sh*C5ar1*, sh*Pf4* or sh*C5ar1+shPf4* CD45⁺ adipocytes (n = 3). Scale bars = 20μm
(j) Quantification of lipid accumulation of Oil red O staining of CD45⁻ brown adipocytes cultured in conditioned media from shNC, sh*C5ar1*, sh*Pf4* or sh*C5ar1+shPf4* CD45⁺ adipocytes (n=6).
(k) Quantification of immunoblotting for UCP1 and PPARγ of adipocyte differentiation of CD45⁻ ASCs cultured in conditioned media from shNC, sh*C5ar1*, sh*Pf4* or sh*C5ar1+shPf4* CD45⁺ adipocytes (n = 3).
Data are represented as mean ± SEM * ≤ 0.05, ** ≤ 0.01, *** ≤ 0.005.

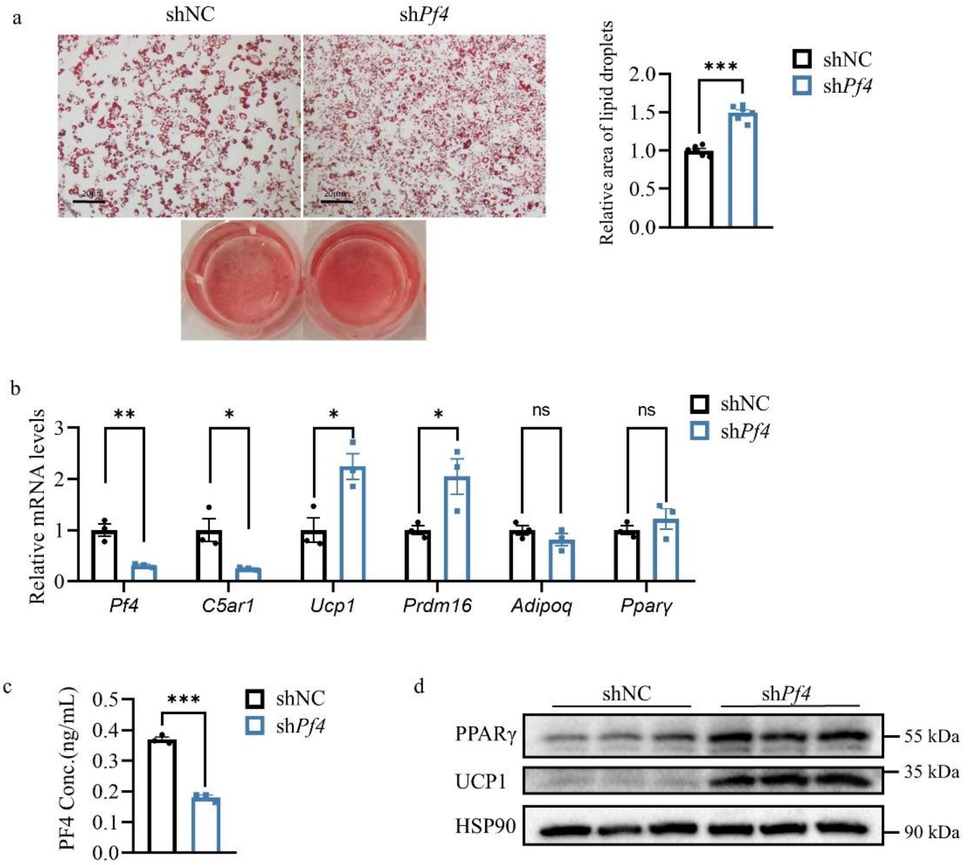

Figure S5. *Pf4* knockdown in CD45⁺ brown adipocytes could increase thermogenic activity, related to Figure 4.

(a) Oil red O staining of shNC and sh*Pf4* CD45⁺ adipocytes. Scale bars = 20μm

(b) The mRNA expression of the indicated genes of shNC and sh*Pf4* CD45⁺ adipocytes (n =3).

(c) The concentration of PF4 in the supernatant of *Pf4* knockdown differentiated CD45⁺ adipocytes compared to the control group (n = 3).

(d) Immunoblotting for UCP1 and PPARγ of *Pf4* knockdown differentiated CD45⁺ adipocytes.

Data are represented as mean ± SEM * ≤ 0.05, ** ≤ 0.01, *** ≤ 0.005.

Table S1. RT-qPCR Primers, Related to Star Methods

Gene	Forward 5' – 3'	Reverse 3' – 5'
Cd45	GACAGAGTTAGTGAATGGAGACC	AAAAGTTCGGAGAGTGTAGGC
Pdgfrα	GTTGCCTTACGACTCCAGATG	TCACAGCCACCTTCATTACAG
Cd34	GTGTTTGCTGATGGTCTTGG	GTCTTTCGGGAATAGCTCTGG
Cd29	GCAACGCATATCTGGAAACTTG	CAAAGTGAAACCCAGCATCC
Pparg	GTGCCAGTTTCGATCCGTAGA	GGCCAGCATCGTGTAGATGA
Fabp4	CACCGAGATTTCCTTCAAACTG	CACGCCTTTCATAACACATTCC
Cd36	GGAGTGCTGGATTAGTGGTTAG	GCTGTGAGCAGACGTATAGAAG
Ucp1	ACTGCCACACCTCCAGTCATT	CTTTGCCTCACTCAGGATTGG
Prdm16	CAGCACGGTGAAGCCATTC	GCGTGCATCCGCTTGTG
Ebf2	GCTGCGGGAACCGGAACGAGA	ACACGACCTGGAACCGCCTCA
Cidea	TGCTCTTCTGTATCGCCCAGT	GCCGTGTTAAGGAATCTGCTG
Cox8b	GAACCATGAAGCCAACGACT	GCGAAGTTCACAGTGGTTCC
Pgc1a	CCCTGCCATTGTTAAGACC	TGCTGCTGTTCCTGTTTTC
Adipoq	TGGAGAGAAGGGAGAGAAAGG	TGAGCGATACACATAAGCGG
Retn	CTGTCCAGTCTATCCTTGCACAC	CAGAAGGCACAGCAGTCTTGA
C5ar1	GTGGCCTGGGTCTTAGCATT	ATCACCCCGGTCACCTGATA
Pf4	GGTCTTGACATGAGCGTCG	CACAGCTAAGATCTCCATCGC
Ccl3	ACACTCTGCAACCAAGTCTTC	AGGAAAATGACACCTGGCTG
Ccl4	AAACCTAACCCCGAGCAAC	CGGGAGGTGTAAGAGAAACAG
Ccl12	CATCAGTCCTCAGGTATTGGC	TTGTGATTCTCCTGTAGCTCTTC
Pltp	CGCAAAGGGCCACTTTTACTA	GCCCCCATCATATAAGAACCAG
36b4	TGACATCGTCTTTAAACCCCG	TGTCTGCTCCCACAATGAAG
Hprt	TCAGTCAACGGGGGACATAAA	GGGGCTGTACTGCTTAACCAG
C3ar1	AATTCCATCTCAGTGTGCTTG	CCTAGCAGTCCCAATAGACAAG
